# Antidepressant-like activity of the neuropeptide Y Y5 receptor antagonist Lu AA33810: behavioral, molecular, and immunohistochemical evidence

**DOI:** 10.1007/s00213-016-4495-3

**Published:** 2016-12-14

**Authors:** Helena Domin, Bernadeta Szewczyk, Bartłomiej Pochwat, Monika Woźniak, Maria Śmiałowska

**Affiliations:** Institute of Pharmacology, Polish Academy of Sciences, Department of Neurobiology, 31-343 Kraków, Smętna street 12, Poland

**Keywords:** Astrocyte, Antidepressant, BDNF, Lu AA33810, GFAP, Neuropeptide Y, Prefrontal cortex, Gliotoxin, Forced swim test

## Abstract

**Rationale:**

It has recently been found that chronic treatment with the highly selective, brain-penetrating Y5 receptor antagonist, Lu AA33810 [N-[[trans-4-[(4,5-dihydro [1] benzothiepino[5,4-d] thiazol-2-yl) amino] cyclohexyl]methyl]-methanesulfonamide], produces antidepressant-like effects in the rat chronic mild stress model.

**Objective:**

In the present study, we investigated the possible antidepressant-like activity of Lu AA33810 in rats subjected to glial ablation in the prefrontal cortex (PFC) by the gliotoxin L-AAA, which is an astroglial degeneration model of depression.

**Results:**

We observed that Lu AA33810 administered intraperitoneally at a single dose of 10 mg/kg both reversed depressive-like behavioral changes in the forced swim test (FST) and prevented degeneration of astrocytes in the mPFC. The mechanism of antidepressant and glioprotective effects of Lu AA33810 has not been studied, so far. We demonstrated the contribution of the noradrenergic rather than the serotonergic pathway to the antidepressant-like action of Lu AA33810 in the FST. Moreover, we found that antidepressant-like effect of Lu AA33810 was connected with the influence on brain-derived neurotrophic factor (BDNF) protein expression. We also demonstrated the antidepressant-like effect of Lu AA33810 in the FST in rats which did not receive the gliotoxin. We found that intracerebroventricular injection of the selective MAPK/ERK inhibitor U0126 (5 μg/2 μl) and the selective PI3K inhibitor LY294002 (10 nmol/2 μl) significantly inhibited the anti-immobility effect of Lu AA33810 in the FST in rats, suggesting that MAPK/ERK and PI3K signaling pathways could be involved in the antidepressant-like effect of Lu AA33810.

**Conclusion:**

O﻿ur results indicate that Lu AA33810 exerts an antidepressant-like effect and suggest the Y5 receptors as a promising target for antidepressant therapy.

## Introduction

Major depressive disorder (MDD), also called major depression, is the commonly occurring mental disease affecting more than 120 million people worldwide (Belmaker and Agam 2008). Despite intensive research in the last 60 years, currently used antidepressant therapies are not efficient enough and depression requires long-term treatment (Thompson et al. 2015). Approximately 30% of patients with depression fail to respond to currently available therapies, which mainly influence monoaminergic systems, therefore, research aimed to find new drugs is still in progress (Prins et al. 2011). The difficulties in efficient antidepressant treatment may be caused by a heterogeneous nature of this mood disorder, associated with different molecular, environmental, and genetic factors. In the last years, studies on depression have shifted from monoamines toward other mechanisms, including glutamatergic neurotransmission (Catena-Dell’Osso et al. 2013).

Glutamate, the major excitatory neurotransmitter in the mammalian brain, is in a balance with gamma-aminobutyric acid (GABA), which is the main inhibitory amino acid neurotransmitter in the brain (Cotman et al. 1981; Lloyd et al. 1986; Wierońska and Pilc 2009). It can be suggested that dysregulation of Glu/GABA is involved in the pathogenesis of depression (Wierońska and Pilc 2009; Hashimoto 2009). An increased level of Glu was found in the brains and cerebrospinal fluid (CSF) of depressed patients (Hashimoto et al. 2007) as well as in their serum (Kim et al. 1982) and plasma (Altamura et al. 1993; Mitani et al. 2006). Furthermore, several studies have shown that the inhibition of glutamatergic neurotransmission was strongly correlated with the therapeutic action of a majority of antidepressant drugs (Paul and Skolnick 2003).

Glial cells, especially astrocytes, play a crucial role in the maintenance of Glu/GABA balance (Anderson and Swanson 2000; Schousboe 2003; Wierońska and Pilc 2009). These cells are a critical structural and functional part of the tripartite synapses, in which they play a direct and interactive role with neurons in synaptic transmission (Araque et al. 1999; Halassa et al. 2007). A number of evidences have shown that the dysfunction of astrocytes may be involved in the pathogenesis of depression. Postmortem studies performed on brains of depressed patients demonstrated that a decrease in the density of glial cells in cortical regions, especially in the prefrontal and cingular areas (Ongür et al. 1998; Rajkowska et al. 1999; Rajkowska 2000; Rajkowska et al. 2001; Cotter et al. 2001, 2002; Gittins and Harrison 2011) and in the hippocampus (Cobb et al. 2016) was one of the most consistent findings. These decreases were associated with a reduced level of astrocytic markers, such as glial fibrillary acidic protein (GFAP) (Miguel-Hidalgo et al. 2000) and glutamine synthetase (Choudary et al. 2005). It is interesting that a reduction in the number of astrocytes in the prefrontal cortex (PFC) was also found in rats exposed to chronic unpredictable stress, which is an animal model of depression (Banasr and Duman 2008; Banasr et al. 2010). Those authors showed that astrocytic degeneration in the PFC provoked by gliotoxin L-alpha-aminoadipic acid (L-AAA) induced depressive-like behavior just like chronic stress (Banasr and Duman 2008), which supports the hypothesis that ablation of glia cells may contribute to development of depression (Banasr et al. 2010). This hypothesis was also confirmed by our previous study, in which microinjection of L-AAA into the rat PFC produced depressive-like behavior observed in the forced swim test (FST), and this effect was reversed by the classical antidepressant imipramine and mGluR5 antagonist 3-[(methyl-1,3-thiazol-4-yl)ethynyl]-pyridine (MTEP), a compound with anxiolytic and antidepressant-like properties (Domin et al. 2014). Moreover, biochemical and immunohistochemical studies showed that MTEP also prevented the astrocytic degeneration in this model of depression (Domin et al. 2014).

It was postulated that neuropeptides, especially NPY, might play a significant role in the pathophysiology of depression (Morales-Medina et al. 2010; Morales-Medina et al. 2012a; Kormos and Gaszner 2013). NPY is a 36-amino acid peptide that is widely distributed in the mammalian central nervous system (CNS) (Chronwall et al. 1985; Gray and Morley 1986). In the forebrain, NPY is present on GABAergic interneurons, where it is involved in the inhibition of the release of other neurotransmitters, e.g., glutamate (Greber et al. 1994; Silva et al. 2005). As a neurotransmitter and neuromodulator, NPY activates the specific membrane bound G-protein-coupled receptors (GPCRs) denoted as Y1, Y2, Y3, Y4, Y5, and Y6 (Michel et al. 1998). All these receptor subtypes mediate the NPY’s biological responses via the G_αi_ signaling pathway (Michel 1991; Dumont et al. 1992). There are a number of data indicating that NPY and its receptor ligands produced antidepressant-like effects in animal models of depression (Stogner and Holmes 2000; Redrobe et al. 2002; Ishida et al. 2007; Walker et al. 2009; Packiarajan et al. 2011; Morales-Medina et al. 2012b; Desai et al. 2014). Furthermore, significant changes in NPY-like immunoreactivity (NPY-ir) were observed not only in animal models of depressive disorders (Jiménez-Vasquez et al., 2000a,2000b; Wu et al. 2011) but also in depressed patients who presented reduced levels of NPY in the cerebrospinal fluid and plasma in several studies (Widerlöv et al. 1988; Westrin et al. 1999; Heilig et al. 2004; Soleimani et al. 2014). The above-mentioned results showed that among the NPY receptors, the Y1, Y2, and Y5 receptors (YR) were involved in depression-related disorders. Moreover, the role of Y4R in depression has also been postulated (Painsipp et al. 2008; Tasan et al. 2009). However, despite the promising results indicating that Y receptors may be hopeful targets for antidepressant therapy, the potential therapeutic use of the YR modulators, agonists and antagonists, has not been clinically validated yet.

Recently, it has been found that a highly selective and potent Y5R antagonist, [*N*-[[trans-4-[(4,5-dihydro[1] benzothiepino [5,4-d]thiazol-2-yl) amino] cyclohexyl] methyl]-methanesulfonamide] Lu AA33810, produced antidepressant-like effects in the rat chronic mild stress model and anxiolytic-like effects in the social interaction test (Walker et al. 2009; Packiarajan et al. 2011). This compound could easily cross the blood-brain barrier and was effective after oral and peripheral administration which may be important from the point of view of its possible future clinical applications. To further investigate the role of Y5 receptors in depression-related disorders, in the present study, we examined a possible antidepressant-like activity of Lu AA33810 in rats subjected to glial ablation in the PFC by the gliotoxin L-AAA, as a useful astroglial degeneration model of depression. We tried to find out whether this Y5R antagonist may counteract the effects of astrocyte ablation on behavioral, biochemical, and immunohistochemical parameters. Since no studies identified the signaling pathways engaged in the antidepressant-like effect of Lu AA33810, in the present study, we investigated whether its antidepressant-like effect is connected with an influence on brain-derived neurotrophic factor (BDNF) protein expression, as its role in the therapeutic effect of antidepressants has been postulated (Schmidt et al. 2008; Autry and Monteggia 2012). Moreover, a growing body of data suggests that mitogen-activated protein kinase/extracellular signal-regulated kinase (MAPK/ERK) and phosphatidylinositol 3-kinase (PI3K) signaling pathways are implicated in the pathophysiology of depression and in the antidepressant-like effect of different compounds (Zeni et al. 2012; Di Benedetto et al. 2013), therefore, we examined an involvement of these pathways in antidepressant-like effect of Lu AAA33810 in the FST in rats.

## Material and methods

### Animals

The experiments were performed on male Spraque-Dawley rats (Charles River, Germany) weighing about 250–300 g. The animals were maintained under standard laboratory conditions of lighting (light phase: 7:00–19:00) and temperature (19–21 °C). The rats were age-matched and were housed five to a cage with free access to water and food. All manipulations were performed between 8:00–14.00. All procedures were conducted according to the guidelines of the National Institutes of Health Animal Care and Use Committee and were approved by the Ethics Committee of the Institute of Pharmacology, Polish Academy of Sciences in Krakow. Every effort was made to minimize animal suffering and to reduce the number of animals used.

### Cannulae implantation

For experiments involving a central administration of compounds, rats were anesthetized with ketamine (75 mg/kg i.m.) and xylazine (10 mg/kg i.m.) and, stereotaxically, bilaterally implanted with guide cannulae aimed at the medial prefrontal cortex (mPFC) region (AP = +3.2 mm, L = +1.0 mm from the Bregma, H = −3.5 mm from the skull) or into the brain ventricles (*AP* = −0.4 mm, *L* = +1.5 mm from the Bregma, *H* = −4.6 mm from the skull) (Paxinos and Watson 1986). The guide cannulae (23-gauge stainless steel tubing), were secured by dental cement and anchored to the skull by two stainless steel screws. In order to prevent clogging, stainless steel stylets were placed in the guide cannulae and left until the animals were microinjected. Seven days later, the rats were subjected to behavioral testing and the injections of the drugs into the brain were made with Hamilton micro syringes connected via polyethylene tubing to two stainless steel needles. Solutions were administered bilaterally for 60 s. The injection needles were kept in place for an additional 60 s before they were removed and replaced with a stylet.

### Drug administration

The following drugs were used: L-alpha-aminoadipic acid (L-AAA) (Sigma-Aldrich Chemie GmbH, Germany); [*N*-[[trans-4-[(4,5-dihydro[1] benzothiepino [5,4-d]thiazol-2-yl) amino] cyclohexyl] methyl]-methanesulfonamide] Lu AA33810; 1,4-diamino-2,3-dicyano-1,4-bis[2-aminophenylthio] butadiene (U0126)—n inhibitor of MAPK/ERK; and 2-(4-morpholino)-8-phenyl-4H-1-benzopyran-4-one (LY294002)—an inhibitor of PI3K (Tocris Bioscience, UK). In order to evoke gliotoxic effect, on the 1st and 2nd day of the experiment, the rats were bilaterally microinjected into the mPFC with the astrocytic toxin L-AAA. L-AAA was freshly dissolved in 0.1 M phosphate buffer, pH 7.4, and was microinjected at the dose of 100 μg/2 μl. Control rats were bilaterally injected with a vehicle according to the same schedule. Afterwards, depressive-like behavior was assessed by the FST 72 h (on day 5) after the second gliotoxin or vehicle administration. The dose of L-AAA and the schedule of treatment were chosen on the basis of our previous study (Domin et al. 2014). In order to assess antidepressant-like activity of the Y5R antagonist, the rats were treated with Lu AA33810 in a single dose of 10 mg/kg, 60 min before the FST. Lu AA33810 was dispersed in 0.5% methylcellulose and was administered intraperitoneally (*i.p.)* in a volume of 1 ml/kg. Control rats received vehicle according to the same schedule. In the next part of the study, to examine the involvement of MAPK/ERK and PI3-K signaling pathways in the antidepressant-like effect of Lu AA33810, we used the inhibitors of these intracellular pathways, U0126 and LY294002, respectively. U0126 (5 μg/2 μl) and LY294002 (10 nmol/2 μl) were dissolved in 0.1 M phosphate buffer, pH 7.4, and were administered by the intracerebroventricular (*i.c.v.*) route (1 μl/site) 15 min before Lu AA33810 (10 mg/kg) in rats which did not receive the gliotoxin. Test FST was performed 1 h after the single administration of Lu AA33810. The doses of the used drugs were selected on the basis of literature data for in vivo treatments (for Lu AA33810 from Walker et al. (2009) and Packiarajan et al. (2011); for U0126 and LY294002 from Zeni et al. (2012) and Manosso et al. (2015)). The same protocol of the administration of the above-mentioned drugs was used to evaluate their influence on the locomotor activity.

### Forced swim test

Procedure of the test was described in the paper by Szewczyk et al. (2009). Briefly, the rats were placed in glass cylinders (height 40 cm, diameter 20 cm) containing 15 cm of water, maintained at 25 °C. Generally, FST consists of two swim sessions: an initial 15-min pretest followed 24 h later by a 5-min test. After both sessions, rats were removed from cylinders, dried, and returned to their home cages. During the 5-min test session, three different behaviors were scored according to Detke et al. (1995): (1) immobility—rats remained floating passively in the water, (2) swimming—rats were making active swimming motions, and (3) climbing—rats were making active movements in and out of the water with their forepaws, directed against the walls.

### Locomotor activity

Following the FST, the locomotor activity of rats was measured. Rats were placed individually in Opto-Varimex cages (Columbus Instruments, USA) connected on-line to a compatible IBM-PC. The behavior of the rats was analyzed using an auto-track software (Columbus Instruments, USA). Each cage (43 × 44 × 25 cm) had a 15 × 15 array of infrared emitter photocell beams located 3 cm from the floor surface. The number of light beams interrupted by an animal was calculated at 5-min intervals and presented as the distance traveled in centimeters.

### Western blot analysis

On the eighth day (144 h after the second gliotoxin injection), the rats were decapitated and their brains were collected. The PFC was rapidly taken by cutting the anterior part of the forebrain at the level of bregma 2.20 mm (Paxinos and Watson 1986). The tissue were frozen on dry ice and stored at −80 °C until biochemical experiments. This step started from tissue homogenization that was conducted in a 2% solution of sodium dodecyl sulfate (SDS). The homogenates were then denatured at 95 °C for 10 min and centrifuged for 5 min at 10,000 rpm at 4 °C, and the supernatant was collected. The protein concentration was determined using a Pierce BCA Protein Assay Kit (Thermo Scientific, USA). Next, 30 μg of protein were separated by 10% SDS-polyacrylamide gel electrophoresis and transferred to nitrocellulose membranes (Invitrogen, Paisley, UK). Non-specific binding was blocked for 1 h in 1% blocking solution [BM Chemiluminescence Western Blotting Kit (Mouse/Rabbit), Roche, Switzerland]. After blocking, the membranes were incubated overnight at 4 °C with the respective primary antibodies: mouse monoclonal anti-GFAP antibody (1:1000, Millipore, Germany), mouse monoclonal anti-GAD67 antibody (1:1000, Millipore, Germany), rabbit polyclonal anti-BDNF antibody (1:500, Santa Cruz Biotechnology, USA), rabbit polyclonal anti-NPY antibody (1:500, Sigma-Aldrich, USA), rabbit polyclonal anti-NPY5R antibody (1:1000, Abcam, USA), and rabbit polyclonal anti-TrkB antibody (1:1000, Abcam, USA). After incubation, the membranes were washed three times for 10 min in Tris-buffered saline with Tween (TBS-T) and incubated for 30 min with anti-mouse/ant-rabbit-IgG-peroxidase-conjugated antibodies (BM Chemiluminescence Western Blotting Kit, mouse/rabbit, Roche; diluted 1:7000)). After incubation with secondary antibodies, the membranes were washed three times for 10 min with TBS-T. In the last step, the blots were incubated with a detection reagent (Roche). The signal from the tested proteins was visualized using the Fuji-Las 1000 system. The density of each protein band was quantified using the Fuji Image Gauge v.4.0 software. To confirm the equal transfer and loading of the samples on the gel, the blots were incubated with rabbit polyclonal anti-GAPDH antibody (1:1000, Bios). The final results are presented as the ratio of the optical density of particular proteins to the optical density of GAPDH.

### Histology and immunohistochemistry

On the eighth day of the experiment, the brains were collected for histological and immunohistochemical analysis, as described previously (Domin et al. 2014). Briefly, the rats deeply anesthetized by pentobarbital were perfused through the ascending aorta with physiological saline, followed by a cold 4% paraformaldehyde (PF) in 0.1 M sodium phosphate buffer (PBS), pH 7.4. Then, their brains were taken out, postfixed in cold buffered PF for 3 h, and cryoprotected in a 20% sucrose solution in PBS for at least 5 days at 4 °C. Next, the brains were frozen on dry ice and cut at 40 μm frontal sections, at levels containing mPFC (between bregma 4.70 to 1.70 mm) (Paxinos and Watson 1986). Sections were collected in PBS and every sixth section was taken for immunohistochemistry. First, the sections were permeabilized with 0.1 M PBS containing 0.2% Triton X-100 (PBS-TX-100) for 30 min after which the blocking was performed in the presence of 5% normal horse serum (NHS) or 5% normal goat serum (NGS) in PBS at room temperature (RT) for 30 min. Next, the sections were incubated (48 h at 4 °C) with the following primary antibodies: mouse monoclonal anti-GFAP antibody (1:800, Millipore, Germany) and rabbit polyclonal anti-BDNF antibody (1:100, Santa Cruz Biotechnology, USA). Anti-GFAP antibody was diluted in 0.2% PBS-TX-100 and 3% NHS and anti-BDNF antibodies was diluted in 0.2% PBS-TX-100 and 3% NGS. After that time, the sections were washed in PBS and incubated for 60 min at RT in the secondary antibodies: anti-mouse IgG (1:500) or anti-rabbit IgG (1:500). Next, the sections were processed by an avidine-biotin peroxidase complex method using an ABC-peroxidase kit (Vector Lab) and diaminobenzidine (DAB) as a chromogen. Additionally, in order to verify the injection sites, the sections adjacent to those labeled with the GFAP or BDNF antibodies were stained with 1% cresyl violet. In the last step, the immunostained sections were washed in Tris and were mounted on slides, dried, dehydrated, cleared in xylene, cover-slipped with Permount, and analyzed under a light microscope Nikon Eclipse E600 (Nikon, Japan).

### Immunofluorescence staining

In order to visualize Y5R- and GAD67-immunoreactive (ir) neurons, the sections were blocked with 0.01 M PBS containing 0.2% Triton X-100 and 5% normal donkey serum at RT for 1 h. After that time, the sections were incubated (48 h at 4 °C) with the following primary antibodies: goat polyclonal anti-NPY Y5R antibody (1:50, Santa Cruz Biotechnology, USA) and mouse monoclonal anti-GAD67 antibody (1:1000, Millipore, Germany) diluted in the blocking buffer. Next, the sections were washed in PBS and incubated overnight at 4 °C in the following mixture of secondary antibodies: Alexa Fluor® 488-labeled donkey anti-goat IgG and Alexa Fluor® 555-labeled donkey anti-mouse IgG (Invitrogen, Poland) diluted in 0.2% PBS-TX-100 and 3% normal donkey serum. After that time, sections were washed in PBS and mounted with a coverslip overlay. For double labeling, the slices were analyzed with a fluorescent microscope Nikon Eclipse E600 (Nikon, Japan), which was equipped with black-white camera (Leica Microsystems CMS, GmbH Germany) connected to a computer equipped with Leica Application Suite (LAS) version 4.5 software at excitation wavelengths of 465–495 nm (Alexa Fluor® 488) and 540–580 nm (Alexa Fluor® 555).

### Stereological counting of GFAP-ir cells within the mPFC

GFAP-ir cells in the mPFC were counted stereologically using a light microscope (Leica, DMLB; Leica, Denmark) equipped with a projecting camera (Basler Vision Technologies, Germany) and a microscope stage connected to an xyz stepper (PRIOR ProScan) controlled by a computer using Visiopharm New CAST software (Visiopharm, Denmark) as described previously (Domin et al. 2014).

Systemic uniform, random sampling was used to select the sections for the analysis. For stereological assessment, cell counts were performed within the contours of the mPFC (AP = 4.70 to 1.70 mm from bregma) (Paxinos and Watson 1986) in 10–12 sections at 240-μm intervals. The analyzed region was outlined under lower magnification (×5), while the number of GFAP-positive cells was estimated under ×63 magnification using a randomized meander sampling and the optical dissector methods according to the formula: *N* = Σ*Q* × *V* (ref)/*v* (dis) × Σ*P*, where Σ*Q* is a total count of GFAP-ir astrocytes in the uniformly sampled dissectors, *V* (ref)—the total volume of the mPFC, *v* (dis)—the total volume of the dissector (Sterio 1984), and Σ*P*—the total number of all dissector points. The total volume of the mPFC *V* (ref) was assessed using the Cavalieri’s rule (Gundersen et al. 1999) according to the formula: *V* (ref) = *t* × *a (p)* × Σ*P*, where *t* is the known distance between the sampled sections, *a (p)* is the area associated with each point of a grid, and Σ*P* is the total number of counted points over all sections from one rat. The area of counting frame was 5559.37 μm^2^ and covered 20% of the screen area. The dissector height was 20 μm and a sampling grid was 333.45 × 333.45 μm (111,188.9 μm^2^). The efficacy of sampling was optimized by the estimation of the coefficient of error (CE) as previously described (West et al. 1996).

### Counting of BDNF-ir cells within the mPFC

The number of BDNF-ir cells in the rat mPFC was quantified within four stained sections from each brain (AP = 4.20 to 2.20 mm from bregma) (Paxinos and Watson 1986) using a light microscope (Leica, DMLB; Leica, Denmark). Only distinct brown cells clearly visible above a background were considered to be immunopositive. The analyzed area was outlined under lower magnification (×5), while the number of BDNF-positive cells was counted under ×63 magnification using a randomized meander sampling and the optical dissector methods using the Visiopharm New CAST software (Visiopharm, Denmark). The area of counting frame was 5455.76 μm^2^. The dissector height was 20 μm and a sampling grid was 330.33 × 330.33 μm (109,117,9 μm^2^).

### Statistical analysis

All results are presented as the means ± SEM. Behavioral data, immunoblots, and immunohistochemical analysis were evaluated using the two-way ANOVA (analysis of variance), followed by the Newman-Keuls (behavioral results) or Bonferroni (biochemical and immunohistochemical results) multiple comparison post hoc tests (GraphPad Software, San Diego CA, USA). The differences were considered to be statistically significant at *p* < 0.05.

## Results

### Behavioral studies

#### The effect of combined administration of L-AAA and Lu AA33810 in the FST in rats

**Fig. 1:**
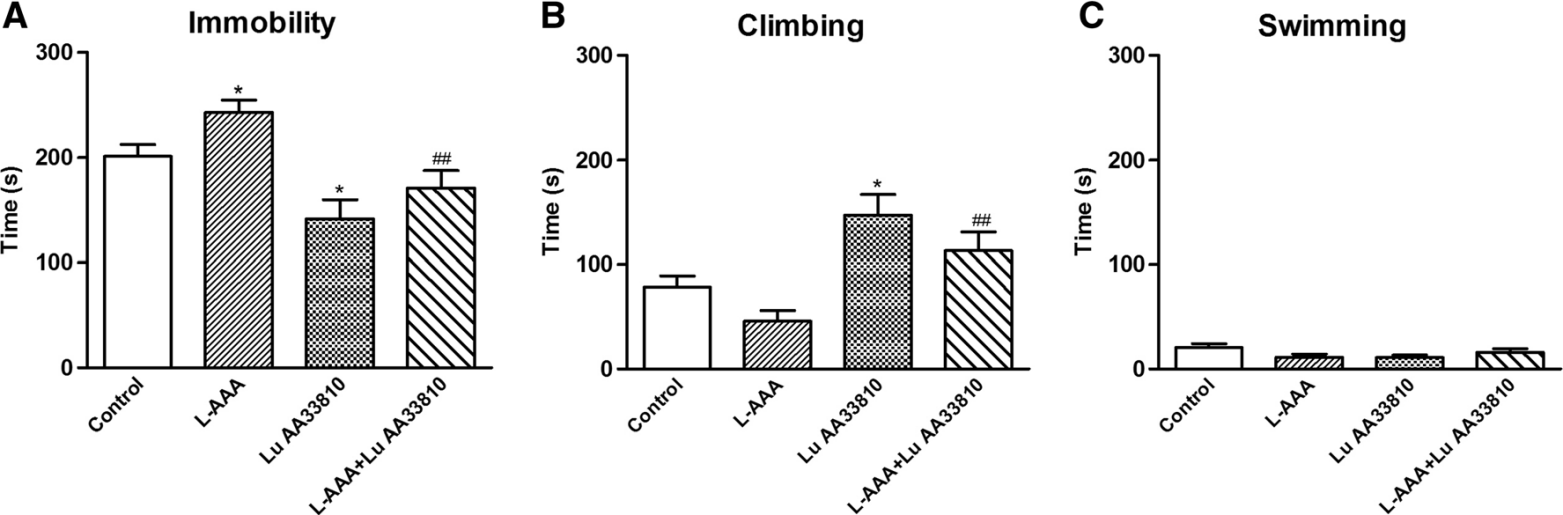
The effect of L-AAA and Lu AA33810 administered alone or in combination on the immobility (**a**), climbing (**b**), and swimming time (**c**) in the FST in rats. L-AAA was administered twice into the rat mPFC at a dose of 100 μg/2 μl, on the first and second day of the experiment and the FST was performed 72 h after the second toxin injection. Lu AA33810, 10 mg/kg, i.p., was given 60 min before the FST. The obtained data were presented as the means ± SEM (*n* = 6–14 rats per group) and evaluated by two-way ANOVA followed by the Newman-Keuls multiple comparison test. *p < 0.05 vs. control; ^##^ *p* < 0.01 vs. L-AAA-treated group

To investigate the possible antidepressant-like activity of Lu AA33810 in rats subjected to glial ablation, we examined the effect of L-AAA and Lu AA33810 in the FST. Compared with the control group, the immobility time increased significantly in L-AAA-treated rats (100 μg/2 μl) (*p* < 0.05, Fig. 1a), indicating that L-AAA-induced, depressive-like behavioral changes in the FST in rats. L-AAA decreased also the climbing time; however, this effect was not significant (Fig. 1b). L-AAA had no influence on the swimming time of rats (Fig. 1c). Lu AA33810 (10 mg/kg) given alone significantly decreased the immobility time (*p* < 0.05) and significantly increased the climbing time (*p* < 0.05) but had no effect on swimming time of rats when compared to the control group (Fig. 1). Compared with the L-AAA-treated group, Lu AA33810 reversed L-AAA-induced increase in the immobility time (*p* < 0.01) and climbing time (*p* < 0.01) but had no effect on the swimming time (Fig. 1). Results of two-way ANOVA for immobility: [F(1,40) = 5.50, *p* = 0.0240] for L-AAA; [F(1,40) = 18.91, *p* = 0.0001] for Lu AA33810 and [F(1,40) = 0.17, *p* = 0.6820] for L-AAA and Lu AA33810 interaction; for climbing: [F(1,40) = 4.91, *p* = 0.0324] for L-AAA; [F(1,40) = 20.34, *p* = 0.0001] for Lu AA33810 and [F(1,40) = 0.003, *p* = 0.9521] for L-AAA and Lu AA33810 interaction; for swimming: [F(1,40) = 0.25, *p* = 0.6189] for L-AAA; [F(1,40) = 0.37, *p* = 0.5471] for Lu AA33810 and [F(1,40) = 3.30, *p* = 0.0769] for L-AAA and Lu AA33810 interaction.

#### The influence of the inhibitors of MAPK/ERK and PI3K signaling pathways on the antidepressant-like effect of Lu AA33810 in the FST in rats

**Fig. 2:**
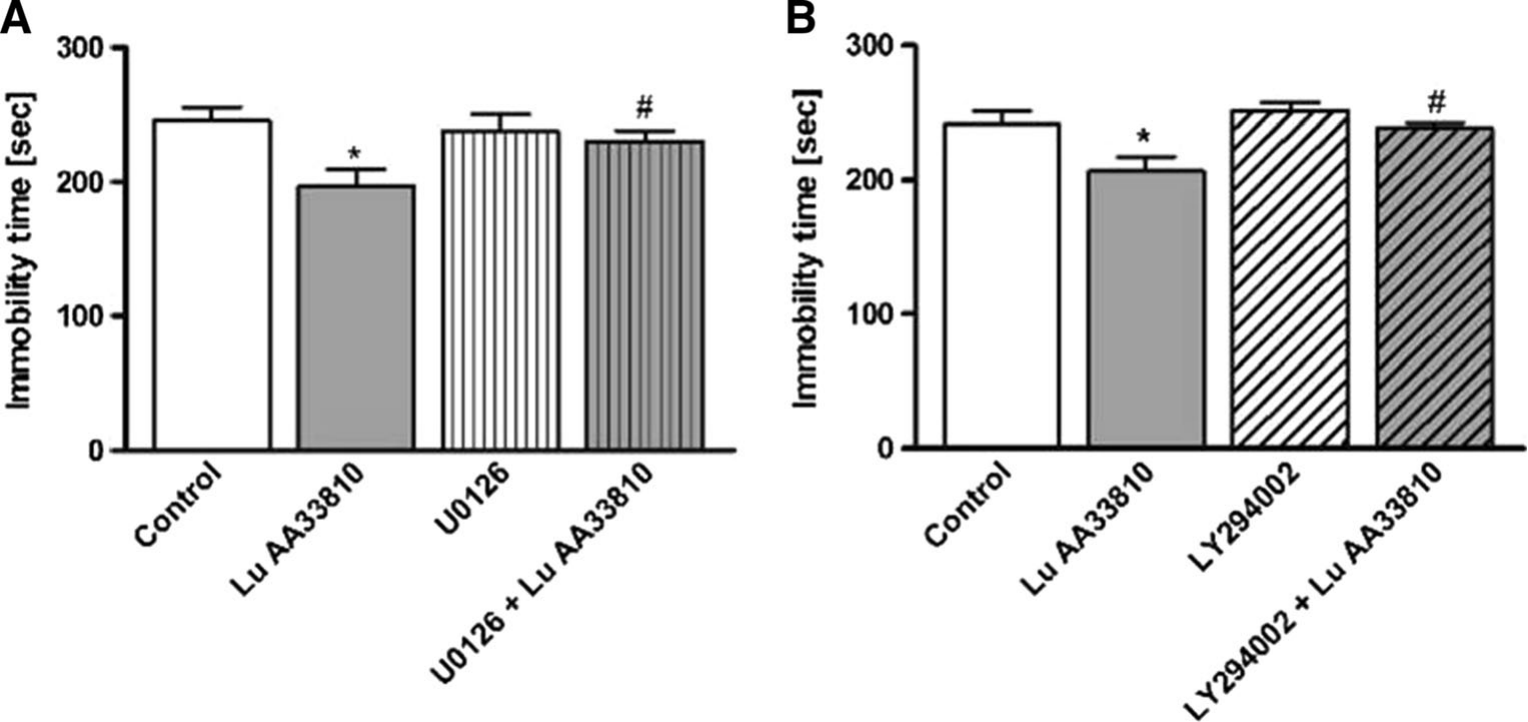
The effects of MAPK/ERK and PI3K inhibitors on the antidepressant-like effect of Lu AA33810 in the FST in rats. Pretreatment with U0126 (5 μg/2 μl/rat, i.c.v.), an MAPK/ERK inhibitor (**a**) and LY294002 (10 nmol/2 μl/rat, i.c.v.) a PI3K inhibitor (**b**) 75 min before the test prevented the anti-immobility effect of Lu AA33810 treatment (10 mg/kg, i.p., 60 min before the test) in the FST in rats. The obtained data were presented as the means ± SEM (*n* = 6–9 rats per group) and evaluated by two-way ANOVA, followed by the Newman-Keuls multiple comparison test. *p < 0.05 vs. control; ^#^ *p* < 0.05 vs. Lu AA33810 treated group

Since previous studies suggested the involvement of MAPK/ERK and PI3K signaling pathways in the antidepressant-like effect of different compounds (Zeni et al. 2012; Di Benedetto et al. 2013), we examined the effect of U0126 (an MAPK/ERK inhibitor) and LY294002 (a PI3K inhibitor) pretreatment on the antidepressant-like effect of Lu AA33810 in the FST in rats. As shown in Fig. 2a, pretreatment with U0126 (5 μg/2 μl) blocked the anti-immobility effect of Lu AA33810 (*p* < 0.05) but did not influence the behavior of rats when given alone (*p* > 0.05). Two-way ANOVA showed a significant effect of Lu AA33810 [F (1, 23) = 6.74 *p* = 0.0161], no effect of U0126 [F(1,23) = 1.45, *p* = 0.2407], and insignificant interaction [F(1,23) = 3.53 *p* = 0.0728]. As shown in Fig. 2b, LY294002 (10 nmol/2 μl) given alone 75 min before the test did not influence the behavior of rats (*p* > 0.05) but significantly blocked the antidepressant-like effect of Lu AA33810 (*p* < 0.05). Two-way ANOVA showed a significant effect of Lu AA33810 [F (1,26) = 7.12 *p* = 0.0130], a significant effect of LY294002 [F(1,26) = 5.41, *p* = 0.0281] and insignificant interaction [F(1,26) = 1.35 *p* = 0.2560].

### The effect of L-AAA, Lu AA33810, and the inhibitors of MAPK/ERK and PI3K signaling pathways on the locomotor activity of rats

L-AAA and Lu AA33810 administered alone or in combined treatment did not significantly change the locomotor activity of rats (Table 1a). No significant changes in the locomotor activity of rats were also observed after administration of Lu AA33810, U0126, LY294002, or the combination of these compounds (Table 1b). Two-way ANOVA demonstrated: for Table 1a: [F(1,33) = 8.45, *p* = 0.006] for LAAA; [F(1,33) = 0.003, *p* = 0.956] for Lu AA33810; and [F(1,33) = 0.93, *p* = 0.341] for L-AAA and Lu AA33810 interaction and for Table 1b: [F(2,29) = 0.674, *p* = 0.517] for U0126 and LY294002 inhibitors; [F(1,29) = 0.199, *p* = 0.658] for Lu AA33810; and [F(2,29) = 0.401, *p* = 0.672] for Lu AA33810 and inhibitors interaction.

**Table 1:** The effect of L-AAA and Lu AA33810 administered alone or in combined treatment (A) and the effect of combined treatment of Lu AA33810 with the inhibitors of MAPK/ERK and PI3K signaling pathways (B) on the locomotor activity of rats

Treatment	Activity counts (5-min test)
A	
Control	100.0 ± 8.61
L-AAA	118.4 ± 9.79
Lu AA33810	90.34 ± 7.04
L-AAA + Lu AA33810	127.0 ± 12.85
B	
Control	100.0 ± 29.9
Lu AA33810	142.4 ± 44.3
U0126	150.3 ± 48.88
Lu AA33810+U0126	121.0 ± 28.75
LY294002	69.36 ± 14.48
Lu AA33810+LY294002	103.1 ± 64.26

The time schedule for the effect of L-AAA, Lu AA33810, and inhibitors is adequate to that used in the FST. The doses of the used compounds are L-AAA (twice at a dose of 100 μg/2 μl), Lu AA33810 (10 mg/kg, i.p.), U0126 (5 μg/2 μl/rat, i.c.v.), and LY294002 (10 nmol/2 μl/rat, i.c.v.). The values represent the mean ± SEM (*n* = 8–16 rats per group) and were evaluated by two-way ANOVA, followed by the Bonferroni multiple comparison test

### Western blot analysis

#### The effect of combined administration of L-AAA and Lu AA33810 on the GFAP protein level in the rat PFC

**Fig. 3:**
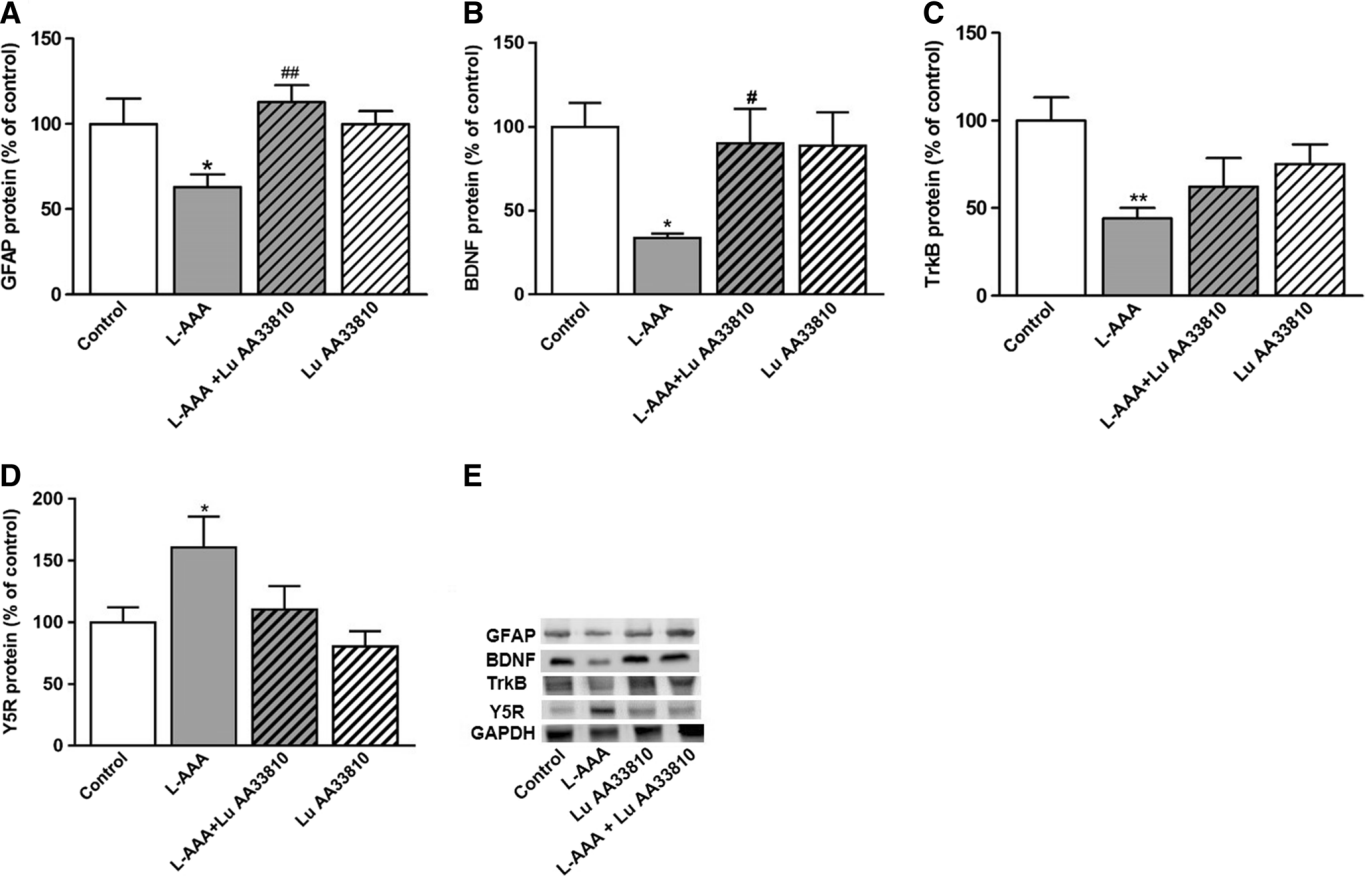
The effect of L-AAA (100 μg/2 μl, twice) and Lu AA33810 (10 mg/kg, i.p.) administered alone or in combination on the protein level of GFAP (**a**), BDNF (**b**), TrkB (**c**), and Y5 receptor (Y5R) (**d**) in the rat PFC determined by Western blot analysis 144 h after the second toxin injection. Data are expressed as % changes vs. control. Immunoblot bands corresponding to GFAP, BDNF, TrkB, Y5R, and GAPDH are seen (**e**). Values represent the mean ± SEM (*n* = 6–10 rats per group) and were evaluated by two-way ANOVA, followed by the Bonferroni multiple comparison test. *p < 0.05, **p < 0.01 vs. control; ^*#*^ *p* < 0.05, ^##^ *p* < 0.01 vs. L-AAA-treated group

Since previous studies showed a correlation between astrocytic degeneration in the mPFC and depression-like behavioral changes (Banasr and Duman 2008; Domin et al. 2014), we examined the effects of L-AAA and Lu AA33810 on the GFAP protein level in the rat PFC. L-AAA administration induced a significant reduction in the GFAP protein level in the rat PFC (*p* < 0.05) (Fig. 3a). Lu AA33810 alone had no effect on the GFAP protein level; however, it antagonized the effect induced by L-AAA (*p* < 0.01) (Fig. 3a). Two-way ANOVA demonstrated no effect of L-AAA [F(1,31) = 1,19 *p* = 0.2835], a significant effect of Lu AA33810 [F(1,31) = 5.06, *p* = 0.0317], and a significant interaction [F(1.31) = 5.09, *p* = 0.0313].

#### The effect of combined administration of L-AAA and Lu AA33810 on the BDNF and TrkB protein levels in the rat PFC

To determine whether BDNF/TrkB signaling pathway is involved in the antidepressant-like effect of Lu AA33810, we examined the effects of L-AAA and Lu AA33810 on the BDNF and TrkB proteins levels in the rat PFC. L-AAA administration induced a significant reduction in the BDNF protein level in the rat PFC (*p* < 0.05) (Fig. 3b). Lu AA33810 alone had no effect on BDNF protein level; however, it antagonized the effect induced by L-AAA (*p* < 0.05) (Fig. 3b). Two-way ANOVA demonstrated a significant effect of L-AAA [F(1,27) = 4.28 *p* = 0.0482], no effect of Lu AA33810 [F(1,27) = 2.08, *p* = 0.1608], and a significant interaction [F(1.27) = 4.52, *p* = 0.0408]. L-AAA administration induced a significant reduction in the TrkB protein level in the rat PFC (*p* < 0.05) (Fig. 3c). Lu AA33810 alone had no effect on TrkB protein level and did not antagonize the effect induced by L-AAA (*p* > 0.05) (Fig. 3c). Two-way ANOVA demonstrated a significant effect of L-AAA [F (1,23) = 8.22 *p* = 0.0087], no effect of Lu AA33810 [F(1,23) = 0.08, *p* = 0.7824], and a significant interaction [F (1.23) = 3.17, *p* = 0.0884].

#### The effect of combined administration of L-AAA and Lu AA33810 on the Y5 receptor protein level in the rat PFC

To determine whether Y5 receptors are implicated in the gliotoxin model of depression, we examined the effect of L-AAA and Lu AA 33810 on the Y5R protein level in the rat PFC. L-AAA administration induced a significant increase in the Y5R protein level in the rat PFC (*p* < 0.05) (Fig. 3d). Lu AA33810 alone had no effect on Y5R protein level and did not antagonize the effect induced by L-AAA (*p* > 0.05) (Fig. 3d). Two-way ANOVA demonstrated a significant effect of L-AAA [F(1,34) = 6.74 *p* = 0.0138], no effect of Lu AA33810 [F(1,34) = 3,97, *p* = 0.0545], and a non-significant interaction [F(1,34) = 0.78, *p* = 0.3847].

### Histological and immunohistochemical analysis

#### The effect of combined administration of L-AAA and Lu AA33810 on the number of GFAP-positive cells in the rat mPFC

**Fig. 4:**
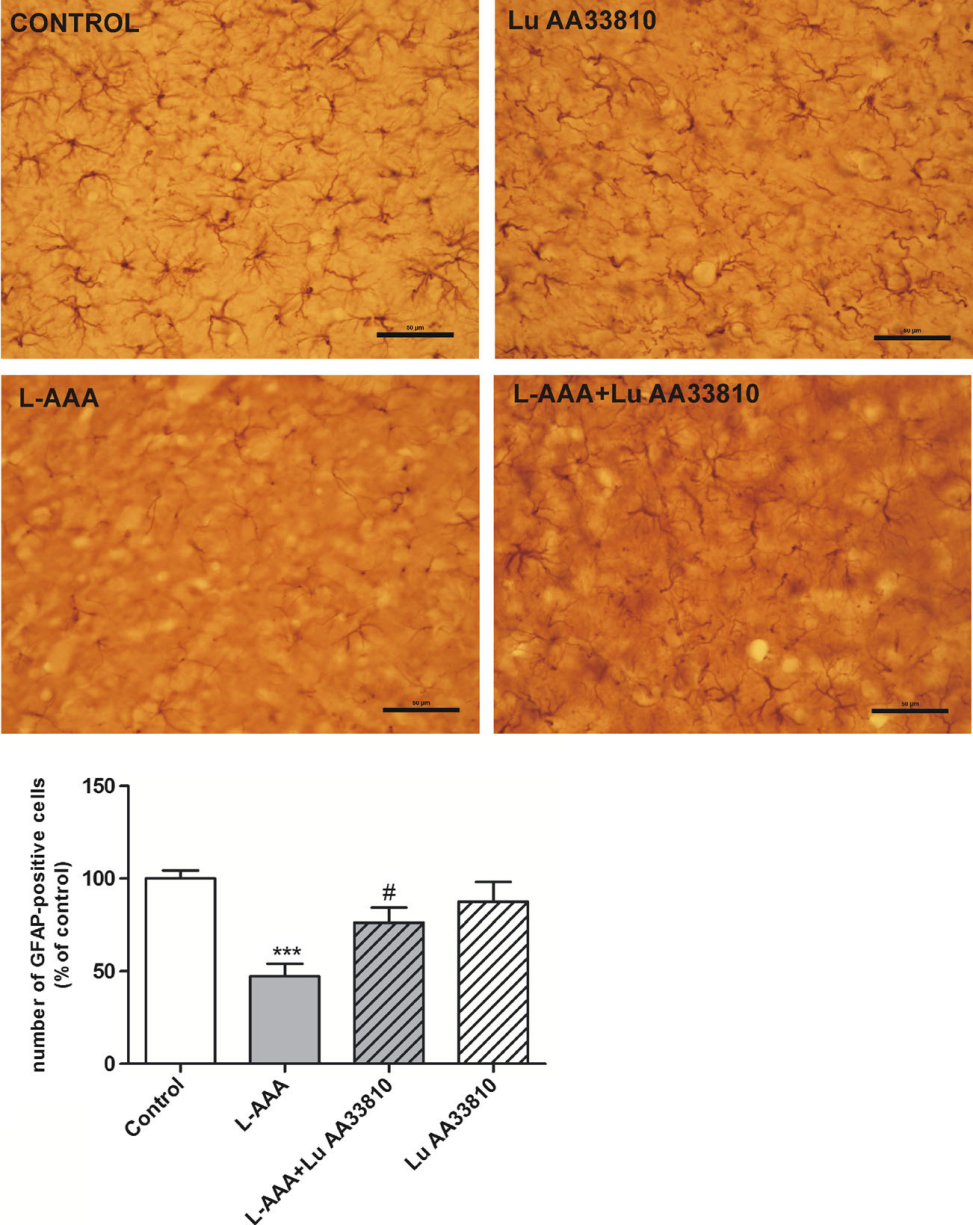
(*Upper panel*) Representative microphotographs of coronal sections of the rat brain, illustrating the effect of L-AAA (100 μg/2 μl, twice) and Lu AA33810 (10 mg/kg, i.p.) administered alone or in combination on the number of GFAP-positive cells in the mPFC. Numerous GFAP-positive astrocytes are seen in the section from a control rat in contrast to few astrocytes in the section from L-AAA injected rat. Moreover, the shrinkage of astrocyte cells and processes is also seen after the gliotoxin. LuAA33810 prevented the decrease in the number of astrocytes and their pathological changes. *Calibration bars* 50 μm. (*Bottom panel*) A histogram showing the number of GFAP-positive cells counted by stereological method. Data are expressed as % changes vs. control. Values represent the mean ± SEM (*n* = 5–6 rats per group) and were evaluated by two-way ANOVA, followed by the Bonferroni multiple comparison test. ***p < 0.001 vs. control; ^#^ *p* < 0.05 vs. L-AAA-treated group

Microscopic observation of sections stained with cresyl violet showed correct cannula placement in the mPFC (data not shown). A reduction of density of GFAP-ir astrocytes was observed in the mPFC of rats injected with L-AAA (Fig. 4, upper panel). Stereological counting demonstrated a significant decrease (by ca. 50%) in the number of GFAP-positive cells in the mPFC after L-AAA compared with control rats (*p* < 0.001) (Fig. 4, bottom panel). Like in Western blot analysis, Lu AA33810 had no effect on the number of GFAP-ir cells, however, it antagonized the effect induced by L-AAA (*p* < 0.05) (Fig. 4, bottom panel). Two-way ANOVA demonstrated no effect of L-AAA [F (1.31) = 1.19 *p* = 0.2835], a significant effect of Lu AA33810 [F(1,31) = 5.06, *p* = 0.0317], and a significant interaction [F(1,31) = 5.09, *p* = 0.0313]. The glioprotective effect of Lu AA33810, which was assessed by stereological counting, was confirmed by the microscopic observation of GFAP-positive cells in the mPFC (Fig. 4, upper panel).

#### The effect of combined administration of L-AAA and Lu AA33810 on the number of BDNF-ir cells in the rat mPFC

**Fig. 5:**
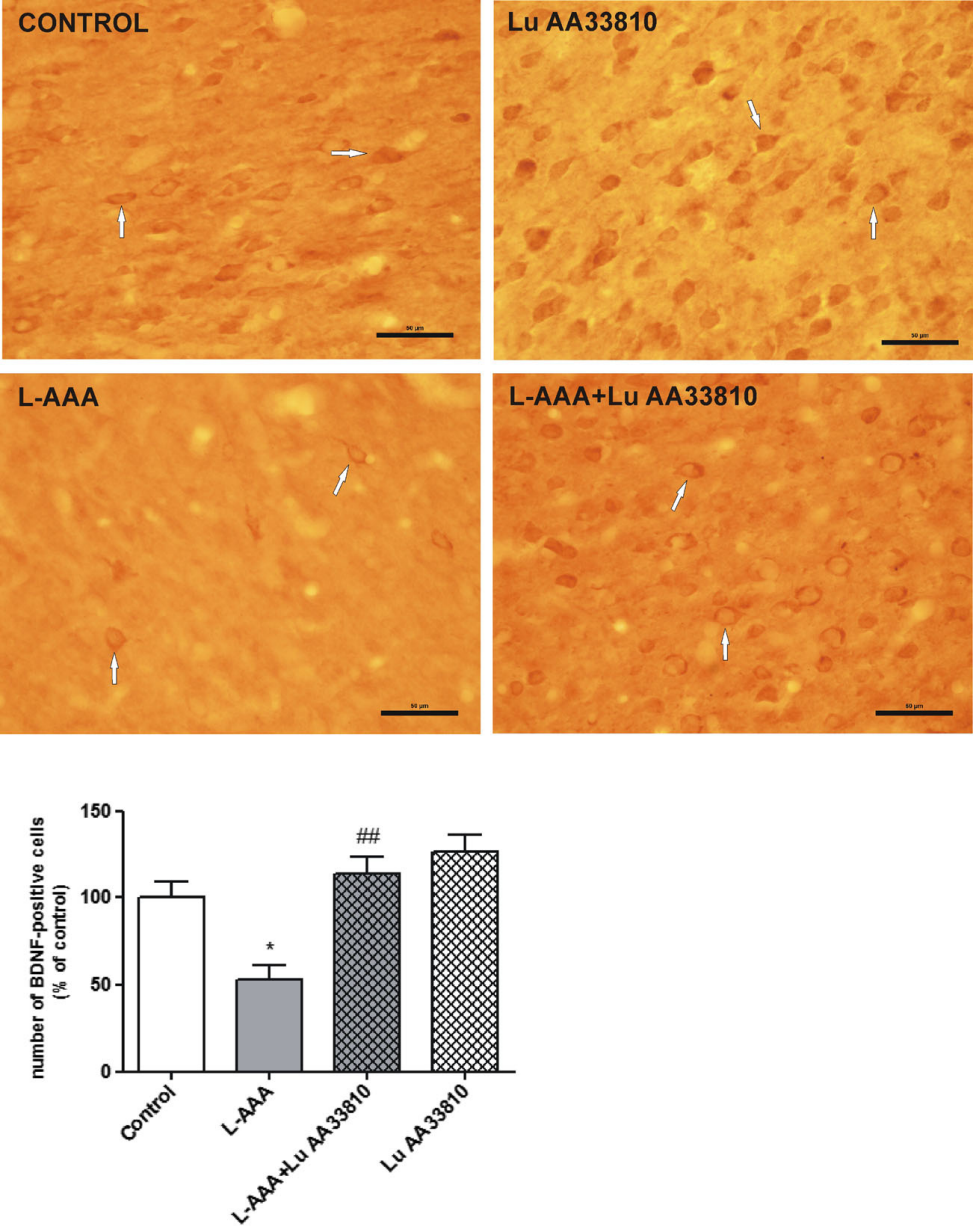
(*Upper panel*) Representative microphotographs of coronal sections of the rat brain showing the expression of BDNF-positive cells in the mPFC of the experimental groups. Many BDNF-positive cells are seen in the control rat. Gliotoxin L-AAA (100 μg/2 μl, twice) induced a strong decrease in the number of BDNF-ir cells, while Lu AA33810 (10 mg/kg, i.p.) reversed this effect. *Arrows* point to some of BDNF-positive cells. *Calibration bars* 50 μm. (*Bottom panel*) A histogram showing the effect of L-AAA and Lu AA33810 administered alone or in combination on the number of BDNF-ir cells in the rat PFC. Data are expressed as % changes vs. control. Values represent the mean ± SEM (*n* = 5–6 rats per group) and were evaluated by two-way ANOVA, followed by the Bonferroni multiple comparison test. *p < 0.05 vs. control; ^##^ *p* < 0.001 vs. L-AAA-treated group

Like the Western blot results, immunohistochemical analysis also showed the decrease in BDNF-ir in the mPFC of rats injected with L-AAA in comparison to control rats, whereas treatment with Lu AA33810 reversed this effect (Fig. 5, upper panel). Compared with the control group, BDNF-ir cells in the mPFC of the L-AAA-treated group decreased to 47% (*p* < 0.05; Fig. 5, bottom panel). Lu AA33810 alone did not evoke any changes in the number of BDNF-ir cells. However, compared with the L-AAA-treated group, there was a significant increase in the number of BDNF-ir cells in the mPFC of the L-AAA+Lu AA33810 group, and the value reached the control value (*p* < 0.01; Fig. 5, bottom panel).

#### The results of double immunofluorescence staining

**Fig. 6:**
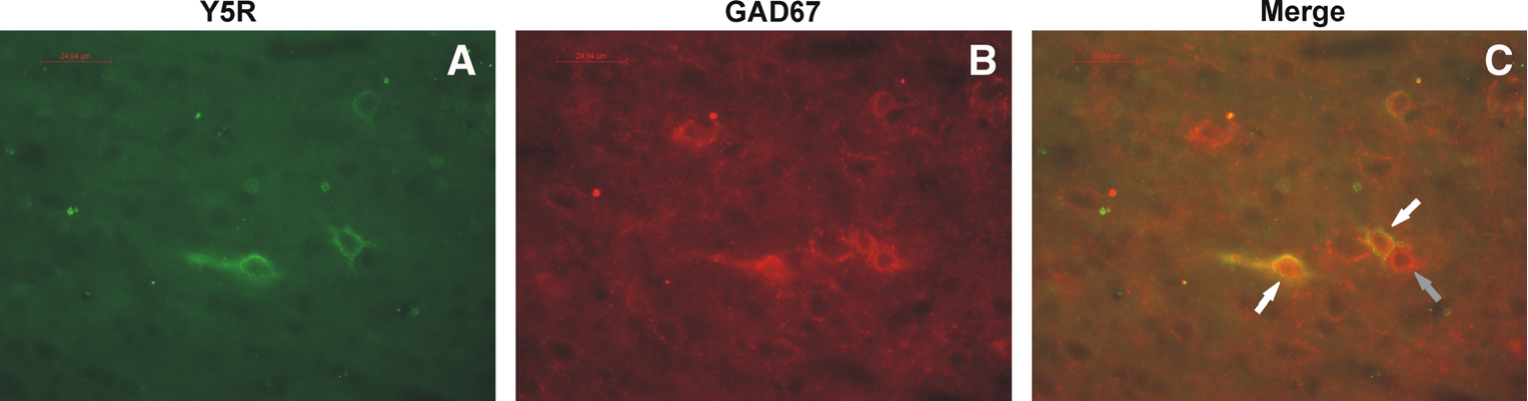
Fluorescence microphotographs of sections of the rat brain mPFC showing immunostaining with anti-NPY Y5R (**a**) and anti-GAD67 (**b**) antibodies. Double-positive neurons are shown by *white arrows* (**c**). *Gray arrow* points to one of GAD67-positive, Y5-negative neuron. *Calibration bars* 25 μm

Microscopic examination of mPFC sections immunostained with anti-NPY Y5R and anti-GAD67 antibodies has shown that Y5R-ir was often found on GAD67-ir neurons (Fig. 6).

## Discussion

The obtained results indicate that the highly selective Y5 receptor antagonist, Lu AA33810, produced antidepressant-like effects in the astroglial ablation model of depression in rats. This compound both reversed depressive-like behavioral changes induced by gliotoxin and prevented degeneration of astrocytes in the PFC. Antidepressant-like activity of Lu AA33810 was examined in the FST which is a preclinical behavioral test widely used for examination of antidepressant-like activity of different drugs (Porsolt et al. 1978; Cryan et al. 2002). A decrease in the immobility time in the FST with no changes in locomotor activity observed by us in the present study after injection of Lu AA33810 both in rats subjected to glial ablation and in rats which did not receive gliotoxin indicates antidepressant potential of this compound. The original FST measured only the immobility time in rodents (Porsolt et al. 1977) whereas the modified FST also measures the swimming time, which is sensitive to serotonergic compounds, and climbing time which is sensitive to drugs with selective effects on noradrenergic transmission (Detke et al. 1995; Cryan et al. 2005). Therefore, the FST may also give us a suggestion on an involvement of serotonergic or noradrenergic pathways in the antidepressant activity of tested compound, since it was found that acceleration of the serotonergic neurotransmission reduced immobility and enhanced the swimming time, while the noradrenergic system activation was associated with a decrease in immobility and improvement of climbing activity (Detke et al. 1995). In the present study, Lu AA33810 both decreased the immobility time and enhanced the climbing behavior but did not change the swimming time. Taking together, the above-mentioned our and others’ results can suggest that the noradrenergic rather than the serotonergic neurotransmission is involved in the antidepressant-like action of Lu AA33810 in the FST. However, further studies are needed to elucidate the involvement of noradrenergic signaling in the antidepressant-like effect of this compound.

The novelty of our present findings is that antidepressant-like effect of Lu AA33810 has been found after a single acute dose (10 mg/kg, i.p.). Other authors studying antidepressant-like effect of Lu AA33810 focused rather on chronic treatment (Walker et al. 2009; Packiarajan et al. 2011). Antidepressant-like effect of Lu AA33819 in rats subjected to chronic mild stress, measured by a normalization of stress-induced decrease in sucrose consumption, was found in response to 10 mg/kg i.p. twice a day in the second week of treatment (Packiarajan et al. 2011) or in response to 3 mg/kg/day i.p. in the third week (Walker et al. 2009). The discrepancies between our and other studies could be explained by the fact that various models of depression (acute glial ablation model of depression vs. chronic mild stress), different strains of rats (Spraque-Dawley vs. Wistar), and different tests for evaluation of potential antidepressant-like effect (FST vs. sucrose consumption test) were used. The studies by Walker et al. (2009) also showed antidepressant-like effect of Lu AA33810 (10 mg/kg/day i.p.) in the FST following chronic treatment, but in contrast to our study, they found such effect of Y5R antagonist in Flinders Sensitive Line (FSL) rats.

The antidepressant efficacy of the Y5R antagonist, Lu AA33810 is in line with observations of many authors who evidenced an important role of NPY in mood disorders (see Introduction). A reduction of the central NPY level and/or function was observed in experimental and clinical depression (Caberlotto et al. 1999; Caberlotto and Hurd 1999), whereas central administration of NPY or Y1R agonist [Leu^31^,Pro^34^]-PYY produced antidepressant-like effects in rats and mice (Stogner and Holmes 2000; Redrobe et al. 2002; Ishida et al. 2007; Morales-Medina et al. 2012a; Desai et al. 2014); moreover, these effects were blocked by Y1R antagonists (Redrobe et al. 2002; Ishida et al. 2007; Desai et al. 2014). On the other hand, antidepressant-like activity of antagonists of Y2 and Y5 receptors was also observed (Walker et al. 2009; Packiarajan et al. 2011; Morales-Medina et al. 2012a, 2012b). It seems to be paradoxical that similar antidepressant-like effect was observed following treatment with NPY and agonist of Y1R or antagonists of Y2 and Y5 receptors. This could be explain by the fact that YRs are differentially distributed in various brain structures, both pre- and postsynaptically, and their activation or inhibition may engage different signaling pathways which underlies the antidepressant-like effects. It seems likely that antidepressant-like effect observed after activation of postsynaptic Y1R was associated with an enhancement of NPY function (Redrobe et al. 2002; Ishida et al. 2007). However, Y2R and Y5R, which are distributed mainly presynaptically, may negatively regulate NPY release (Chen et al. 1997; King et al. 1999), and antagonism of Y2R and/orY5R would thus be expected to increase NPY function in the CNS and may prove to be useful in treating mood disorders. Walker et al. (2009) indicate the antidepressant potential of the Y5R antagonist and its ability to restore NPY levels, which could be partially relevant in FSL rats that not only exhibit a depressed phenotype (Overstreet et al. 2005) but also display an overall reduction in limbic NPY expression (Caberlotto et al. 1999; Jiménez-Vasquez et al. 2000a).

Our present Western blot analysis showed a significant increase in the Y5R protein level in the PFC after administration of gliotoxin (Fig. 3d). The increase in the level of Y5R protein in that astrocyte ablation model may be a compensatory reaction to the postulated decrease in NPY function in depression. Such compensatory increase in receptor level as a consequence of diminution in neurotransmitter function is a generally accepted adaptive response (Kurlan and Shoulson 1982; Pokk et al. 1996). Moreover, Y5R increase may be a result of tissue damage and glutamatergic over-excitation which seems to occur in the mPFC after gliotoxin as an increase in Y5R was found by other authors in the hypothalamus of rats treated with monosodium glutamate (Stricker-Krongrad and Beck 2004), in rat limbic structures during kindling (Kopp et al. 1999), and in human cortical dysplasia in intractable epilepsy (Li et al. 2016). Taken together, the increase in the level of Y5R protein in our astrocyte ablation model suggests that Y5R may play an important role in this model of depression and that the blockade of Y5R has been hypothesized as an indirect way to increase NPY function and improve depression-like behavior.

In our present study, we observed that GABA neurons contained Y5R (Fig. 6), thus this result may suggest that Y5Rs play a role in the regulation of GABAergic neurons in the mPFC. As mentioned above, Y5R activation could inhibit the release of NPY and co-expressed transmitters (Gehlert et al. 2008; Walker et al. 2009), so blockade of these receptors may restore NPY level through inhibition of inhibitory GABA neurons. A possibility of regulation of cortical GABA neurons by Y5Rs was also found by other authors who identified Y5R in the rat cerebral cortex located on the soma and proximal dendrites of GABAergic interneurons (Grove et al. 2000; Bari et al. 2015). Since the model of astrocyte ablation is based on the dysregulation of Glu/GABA balance, it could be suggested that the antidepressant-like effect of Lu AA33810 may be a result of an indirect modulatory action via GABA interneurons which influence Glu/GABA balance.

Our present results have shown that there is a correlation between the astrocytic degeneration in the mPFC and depression-like behavioral changes in our gliotoxin model, because Lu AA33810, given after L-AAA, both prevented astrocytic degeneration (Fig. 4) and exhibited antidepressant-like properties in the FST (Fig. 1). These findings are consistent with our previous results in which we found that L-AAA injected into the mPFC strongly reduced the number of GFAP-positive cells and GFAP protein level in this structure; moreover, the mGlu5 receptor antagonist MTEP, presenting antidepressant-like properties in this model, diminished also the degeneration of astrocytes (Domin et al. 2014). Results of many other authors have also indicated a direct action of antidepressants on astrocytes, modifying their morphology, physiology, and gene expression both in experimental studies and in depressed patients (Liu et al. 2009; Czéh and Di Benedetto 2013; Li et al. 2013). These authors revealed that different antidepressant treatments (e.g., administration of fluoxetine, clozapine, clomipramine, or magnolol) regulated the expression of GFAP as well as other astroglia proteins, such as aquaporin 4 and connexin 43, in the rat frontal cortex and/or hippocampus. Interestingly, in our present study, we observed that Lu AA33810 restored the gliotoxin-induced decrease in GFAP expression after a single dose, whereas other authors have observed the reversal of the glial atrophy after chronic treatment.

The mechanism of antidepressant and glioprotective effects of Lu AA33810 has not been studied so far. Since an important role of BDNF/TrkB signaling pathways in the therapeutic effect of antidepressants has been suggested (Schmidt et al. 2008; Autry and Monteggia 2012), we investigated the cortical BDNF and TrkB protein levels after the acute administration of Lu AA33810 in rats under glial ablation condition. Our Western blot analysis has shown a strong decrease in the protein level of both BDNF and its receptor TrkB in the rat PFC after gliotoxin injection. The decrease in the BDNF protein level was significantly reversed by Lu AA33810, which had antidepressant-like effect in the present gliotoxin model of depression (Fig. 3b). Our Western blot results were confirmed by immunohistochemical analysis showing a reduction in BDNF-ir after gliotoxin, whereas treatment with Lu AA33810 reversed this effect (Fig. 5). These results are in line with numerous clinical findings demonstrating that serum BDNF levels are reduced in depressed patients and then normalized after antidepressant treatment (Brunoni et al. 2008; Dreimüller et al. 2012; Molendijk et al. 2014; Mikoteit et al. 2014; Duclot and Kabbaj 2015; Polyakova et al. 2015). In human postmortem brains collected from suicide victims with major depression, a decrease in TrkB messenger RNA (mRNA) and BDNF mRNA levels was found in the prefrontal cortex and hippocampus (Autry and Monteggia 2012). Therefore, it is postulated that BDNF plays an essential role in the pathophysiology of depression. Similar results were obtained not only in depressed patients but also in experimental studies. Stress and glucocorticoids decreased BDNF protein level and chronic treatment with antidepressants increased BDNF protein and mRNA and TrKB mRNA levels in the rat hippocampus and cortical structures (Altar 1999; Dias et al. 2003; Altar et al. 2003; Larsen et al. 2008). The efficacy of antidepressants was significantly diminished or abolished when BDNF/TrkB signaling pathway was disturbed (Saarelainen et al. 2003; Duclot and Kabbaj 2015). In the present study, we found that Lu AA33810 both revealed antidepressant-like effects and counteracted the astroglial degeneration as well as prevented the decrease in BDNF protein level, suggesting the involvement of the BDNF signaling pathway in its antidepressant-like effect in the present gliotoxin model of depression.

In the present study, we also observed antidepressant-like effect of Lu AA33810 in the FST in rats which did not receive gliotoxin. Considering the possible mechanism underlying its antidepressant-like effect, it should be noted that some authors suggested that MAPK/ERK and PI3K signaling pathways could be involved in the therapeutic effect of different compounds (Zeni et al. 2012; Di Benedetto et al. 2013; Manosso et al. 2015). Our study demonstrated that i.c.v. injection of the selective MAPK/ERK inhibitor U0126 and the selective PI3K inhibitor LY294002 significantly inhibited the anti-immobility effect of Lu AA33810 in the FST in rats (Fig. 2). This result may indicate that the antidepressant potential of Lu AA33810 is most likely due to the activation of the MAPK/ERK and PI3K signaling pathways. The similar effects of U0126 and LY294002 pretreatment were found in the tail suspension test (TST) in mice (Zeni et al. 2012). Those authors demonstrated the involvement of MAPK/ERK and PI3K in the acute antidepressant-like effect of ferulic acid, which is consistent with our results showing the involvement of these signaling pathways in the antidepressant activity of Lu AA33810. The present data are also consistent with our previous findings and results of other authors demonstrating that pretreatment with LY294002 prevented the antidepressant-like effect of compounds in the FST in mice (Budni et al. 2012) and in the FST in rats (Szewczyk et al. 2015).

In conclusion, our results demonstrate that the selective Y5 receptor antagonist, Lu AA33810, produces a rapid antidepressant-like effect which might be mediated by the BDNF as well as by MAPK/ERK and PI3K signaling pathways. We suppose that Y5 receptors may be an attractive target for development of a novel antidepressant therapeutic strategy; however, more data are needed in order to confirm their importance in the treatment of depression (Table 1).

## Abbreviations

BDNF: Brain-derived neurotrophic factor
CNS: Central nervous system
CSF: Cerebrospinal fluid
FST: Forced swim test
GABA: Gamma-aminobutyric acid
GFAP: Glial fibrillary acidic protein
GLT: Glutamate transporter
Glu: Glutamate
GPCRs: G-Protein-coupled receptors
L-AAA: L-Alpha-aminoadipic acid;
Lu AA33810: [*N*-[[Trans-4-[(4,5-dihydro[1]benzothiepino[5,4-d]thiazol-2 yl) amino]cyclohexyl]methyl]-methanesulfonamide]
LY294002: 2-(4-Morpholino)-8-phenyl-4H-1-benzopyran-4-one
MAPK/ERK: Mitogen-activated protein kinase/extracellular signal regulated kinase
MDD: Major depressive disorder
mPFC: Medial prefrontal cortex
MTEP: (3-[(Methyl-1,3-thiazol-4-yl) ethynyl]-pyridine)
NPY: Neuropeptide Y
NPY-ir: NPY-Immunoreactivity
PFC: Prefrontal cortex
PI3K: Phosphatidylinositol 3-kinase
TrkB: Tyrosine receptor kinase B
U0126: 1,4-Diamino-2,3-dicyano-1,4-bis [2-aminophenylthio] butadiene
YR: Y receptors
